# Extracellular Polysaccharide Production by a Novel Osmotolerant Marine Strain of *Alteromonas macleodii* and Its Application towards Biomineralization of Silver

**DOI:** 10.1371/journal.pone.0098798

**Published:** 2014-06-16

**Authors:** Ananya Mehta, Chandni Sidhu, Anil Kumar Pinnaka, Anirban Roy Choudhury

**Affiliations:** CSIR - Institute of Microbial Technology, Council of Scientific and Industrial Research, Chandigarh, India; Dowling College, United States of America

## Abstract

The present study demonstrates exopolysaccharide production by an osmotolerant marine isolate and also describes further application of the purified polysaccharide for production of colloidal suspension of silver nanoparticles with narrow size distribution. Phylogenetic analysis based on 16S r RNA gene sequencing revealed close affinity of the isolate to *Alteromonas macleodii*. Unlike earlier reports, where glucose was used as the carbon source, lactose was found to be the most suitable substrate for polysaccharide production. The strain was capable of producing 23.4 gl^−1^ exopolysaccharide with a productivity of 7.8 gl^−1^ day^−1^ when 15% (w/v) lactose was used as carbon source. Furthermore, the purified polysaccharide was able to produce spherical shaped silver nanoparticles of around 70 nm size as characterized by Uv-vis spectroscopy, Dynamic light scattering and Transmission electron microscopy. These observations suggested possible commercial potential of the isolated strain for production of a polysaccharide which has the capability of synthesizing biocompatible metal nanoparticle.

## Introduction

Exopolysaccharides are long chain biopolymers composed of repeating units of sugar moieties connected via glycosidic linkages and may be obtained from plant as well as microbial sources. In past, terrestrial polymer producing microorganisms have received significant attention in comparison to other natural resources. Although, marine system consists around 70% of the volume of earth, still, this huge diverse environment is relatively less explored. However, in recent time exploitation of marine microflora for isolation of novel bioactive molecules has gained significant interest among researchers worldwide. Marine environment is characterized by extreme physicochemical conditions and harbours unusual microbes with potential of different biotechnological applications. Hence, in order to survive in such conditions marine microbes follow some unique biochemical pathways resulting in the production of novel bioactive compounds including, exopolysaccharides [1], [2]. More importantly, marine microbes tend to have significant osmotic tolerance leading to their capability of polysaccharide production at higher sugar concentration, which is very much desired for development of an economically feasible process for polysaccharide production. Development of suitable application of these biocompounds is also important. Polysaccharides already have found applications in several industrial segments including food, pharmaceuticals and chemical industries [3], [4]. In recent times, biogenic production of metal nanoparticles has also drawn significant interest from scientists. Although, mostly microbes and plant extracts [5], [6] have been utilized for synthesis of metal nanoparticles, however, in both cases the mechanisms for production is ill-defined and the final product may contain several impurities leading towards bio-incompatibility of the produced nanoparticles. Hence, there is a strong need of green, easy and biocompatible technique for metal nanoparticle synthesis. Microbial exopolysaccharides seem to be a promising alternative as they can effectively act as strong reducing as well as stabilizing agent for metal nanoparticle production [7]. Silver nanoparticle has obtained significant attention of researchers due to its potential high value applications in biomedical and pharmaceutical industries. There are few reports available on production of silver nanoparticles (AgNPs) using polysaccharides such as heparin, hyaluronic acid, cellulose, starch, alginic acid etc. [8]–[11]. However most of them are unable to synthesize monodisperse colloidal suspension of AgNPs [12]. Hence, it would be interesting to develop a green process for production of biocompatible stable colloidalsuspension of silver nanoparticle with narrow particle size distribution using polysaccharide.

The present study demonstrates, for the first time, production of polysaccharide using an osmotolerant marine bacterium and subsequent application of that polysaccharide for production of silver nanoparticle. Screening of several marine organisms for their capability of exopolysaccharide production has resulted in isolation of a novel osmotolerant polysaccharide producing strain of *Alteromonas macleodii*. The isolate was able to produce higher quantity of exopolysaccharide when lactose was used as carbon source. The EPS was characterized by using FT-IR spectroscopy. Further, purified EPS was used for the production of biogenic AgNPs. The nanoparticles formed were characterized using Uv-visible spectroscopy, Transmission electron microscopy and Dynamic light scattering.

## Materials and Methods

### Ethics Statement

The samples were taken from Kochi back waters and Arabian Sea and not from a protected land or private property. Hence this does not require any permission for such activities and also does not come under “endangered or protected species”.

### Materials

The media components like Zobell Marine Broth, glucose, maltose, sucrose, lactose were obtained from Hi-Media, Mumbai India. Ethanol and silver nitrate were obtained from Merck India.

### Isolation and cultivation of bacteria

The strains were isolated from samples collected from Arabian Sea and Kochi back water, India. The samples were serially diluted and plated onto marine agar and incubated at 30°C for three days. Unique morphotypes were selected and were purified by subsequent streaking on ZoBell marine agar (ZMA) plates; the purity of the culture was confirmed by observing under phase contrast microscope and also by streaking.

### Screening of isolates for exopolysaccharide production and microbial characterization

Selected bacterial cultures were further screened for EPS production. Inoculum was prepared by transferring bacterial colony grown on ZMA plates to 250 ml conical flask containing 50 ml of Zobell Marine Broth (ZMB) fortified with 5% glucose. Flasks were incubated for 48 hours in an orbital shaker (30°C, 200 rpm). The fermented broth was made cell free by centrifugation at 10,000 rpm for 20 minutes followed by microfiltration through 0.22 µ filter. Cold absolute ethanol was added to supernatant in ratio of 1∶2 (v/v) and kept at 4°C for 24 hours [13] for EPS precipitation. Precipitated EPS was further recovered by removing ethanol and then dried at 80°C overnight. Crude EPS was purified after dialysis by following the protocol described by Chakraborty et al. [14]. Biomass was measured in terms of optical density at 600 nm using corresponding media as blank. Productivity of EPS was expressed as grams of polymer produced per litre of fermentation broth per hour, whereas yield of the polysaccharide was defined as grams of polysaccharide produced per unit biomass.

### Morphological, physiological and biochemical characterization

Colony morphology was studied by observing the strain grown on ZMA for 48 hours at 30°C. Cell morphology and motility were studied by observing actively growing cells under phase contrast microscope. Gram stain reaction was determined using Hi-MEDIA Gram staining kit as per the manufacturer's instructions.

Physiological and biochemical characteristics were determined as described previously [15]. Biochemical and enzymatic characterization of the strain PA2 was performed using Vitek 2 GN kits (bioMerieux) with incubation at 30°C, according to the manufacturer's protocol.

### 16S rRNA gene sequencing and phylogenetic analysis

DNA isolation, 16S rRNA gene amplification and sequencing were done as described previously [16]. The 16S rRNA gene sequence of the isolate was subjected to BLAST sequence similarity search [17] and EzTaxon [18] to identify the nearest related taxa. The 16S rRNA gene sequences of closely related type strains were downloaded from the NCBI database (http://WWW.ncbi.nlm.nih.gov) and aligned using clustal_W program of MEGA version 5.0 [19] and the alignment was corrected manually using BioEdit [20]. Pairwise distances were calculated using the algorithm of kimura-two-parameter [21]. Phylogenetic tree was constructed using the tree making algorithm neighbour-joining method using MEGA 5.0.

### Effect of different Carbon sources

To investigate the effect of different carbon sources over the production of EPS, strain was grown in ZMB fortified with 5% (w/v) of glucose, lactose, maltose and sucrose in each flask. Fermentation was carried out for 96 hours and growth conditions were kept unchanged. After every 24 hours EPS and biomass (in terms of O.D at 600 nm) was measured. All the experiments were carried out in triplicates and averaged data was reported.

### Effect of different concentration of lactose on EPS production

The lactose concentration was varied in the media from 5% (w/v) to 15% (w/v) keeping all other media components and parameters constant. Fermentation was carried out till 96 hours and samples were analysed after every 24 hour interval. EPS and biomass were measured according to the methods described earlier. All experiments were carried out in triplicate and average data were reported.

### Polysaccharide mediated synthesis of silver nanoparticles

In a typical experiment, synthesis of AgNPs was carried out by dissolving 0.05% of EPS into freshly prepared 9 mM aqueous solution of AgNO_3_ in the ratio of 1∶9 (v/v). The mixture was incubated at 30°C in the presence of light. Change in colour of the solution marked the formation of AgNPs. Samples were taken every two hour interval to study the AgNP formation.

### Characterization ofAgNPs

Formation of silver nanoparticles were studied by UV-visible spectroscopy by measuring spectra in the range of 300–800 nm and simultaneously monitoring appearance of the characteristic plasmon resonance of silver nanoparticles using Hitachi double beam spectrophotometer. Further the AgNPs were characterized by studying morphology using Transmission Electron Microscopy (TEM) in a JEOL 2100 TEM and the size distribution of nanoparticles was studied using dynamic light scattering technique as described earlier [22].

### FT-IR analysis of the exopolysaccharide and the nanoparticles formed

FT-IR spectra of the EPS and AgNPs were measured using Perkin Elmer spectrophotometer. In each case sample for analysis was prepared by finely grinding the sample with potassium bromide. Overnight desiccation at 50°C of sample was done under low pressure to remove any moisture content in it. Spectra was recorded over potassium bromide pellets within a range of 4000–400 cm^−1^,16 scans with resolution of 2 cm^−1^ [23], [24].

## Results and Discussion

### Screening, microbial Characterization and Phylogenetic analysis

**Figure 1:**
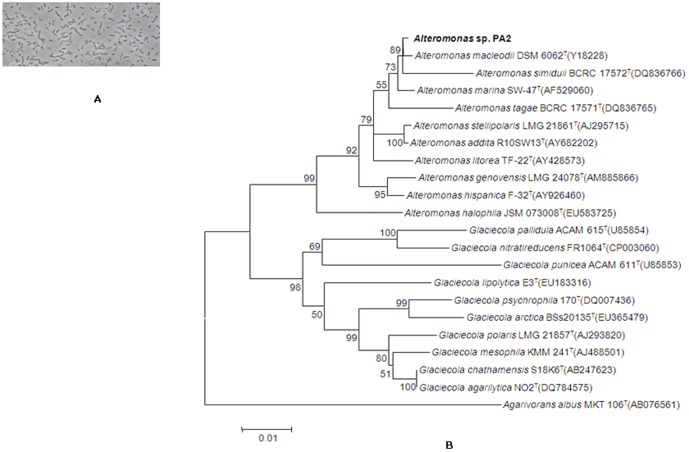
Taxonomic characterization of the producing microbe. (A) Phase contrast microphotograph of the strain *Alteromonas macleodii* PA2. (B) Neighbour-joining tree based on 16S rRNA gene sequences showing the phylogenetic relationship.

Ten cultures were selected on the basis of colony morphology and were screened for the production of EPS (Table 1).Among those 10 strains only one strain, designated as PA2, showed capability of exopolysaccharide production and was further selected for polyphasic taxonomic studies. Cells of the strain PA2 were found to be Gram-stain-negative, rod shaped, non-spore forming and strictly aerobic (Figure 1A). Biochemical and physiological characterization of the selected strain was carried out (Tables S1 and S2 in File S1). 16S rRNA gene sequencing was done for all the ten selected strains and their identity was determined carrying out sequence similarity search using EzTaxon [18]. Among the ten isolates two strains (PA1 and PA5) were related to *Pseudoalteromonas prydzensis*, two strains (PA2 and PA8) were close to *Alteromonas macleodii*, four strains (PA3, PA6, PA7, and PA9) were close to *Vibrio* spp., one strain (PA4) was close to *Aesturaribacter aggregates* (100%) and strain PA10 was close to *Ferrimonasfuttsuensis* (98.34%). Phylogenetic analysis of the strain PA2 based on 16S rRNA gene sequences revealed that it was closely related to the genus *Alteromonas* of the family *Alteromonadaceae* and was having a pairwise sequence similarity of 99.7%. with phylogenetic neighbour *Alteromonas macleodii* (Figure 1B) and this novel strain was designated as *Alteromonas macleodii* PA2.

**Table 1:** Details of the isolates screened for production of exopolysaccharide.

S. No	Strain	Source of Isolation	Organism with closest proximity
1	PA1	Water sample from Arabian sea	*Pseudoalteromonad prydzensis*
2	PA2	Water sample from Arabian sea	*Alteromonas macleodii*
3	PA3	Sediment sample from Kochi back water	*Vibrio azureus*
4	PA4	Water sample from Arabian sea	*Aesturaribacter aggregates*
5	PA5	Water sample from Arabian sea	*Psuedoalteromonas prydzensis*
6	PA6	Water sample from Arabian sea	*Vibrio rotiferianus*
7	PA7	Water sample from Arabian sea	*Vibrio owensii*
8	PA8	Sediment sample from Kochi back water	*Alteromonas macleodii*
9	PA9	Water sample from Arabian sea	Vibrio harveyi
10	PA10	Sediment sample from Kochi back water	*Ferrimonas futtsuensis*

### Effect of different carbon sources

**Figure 2:**
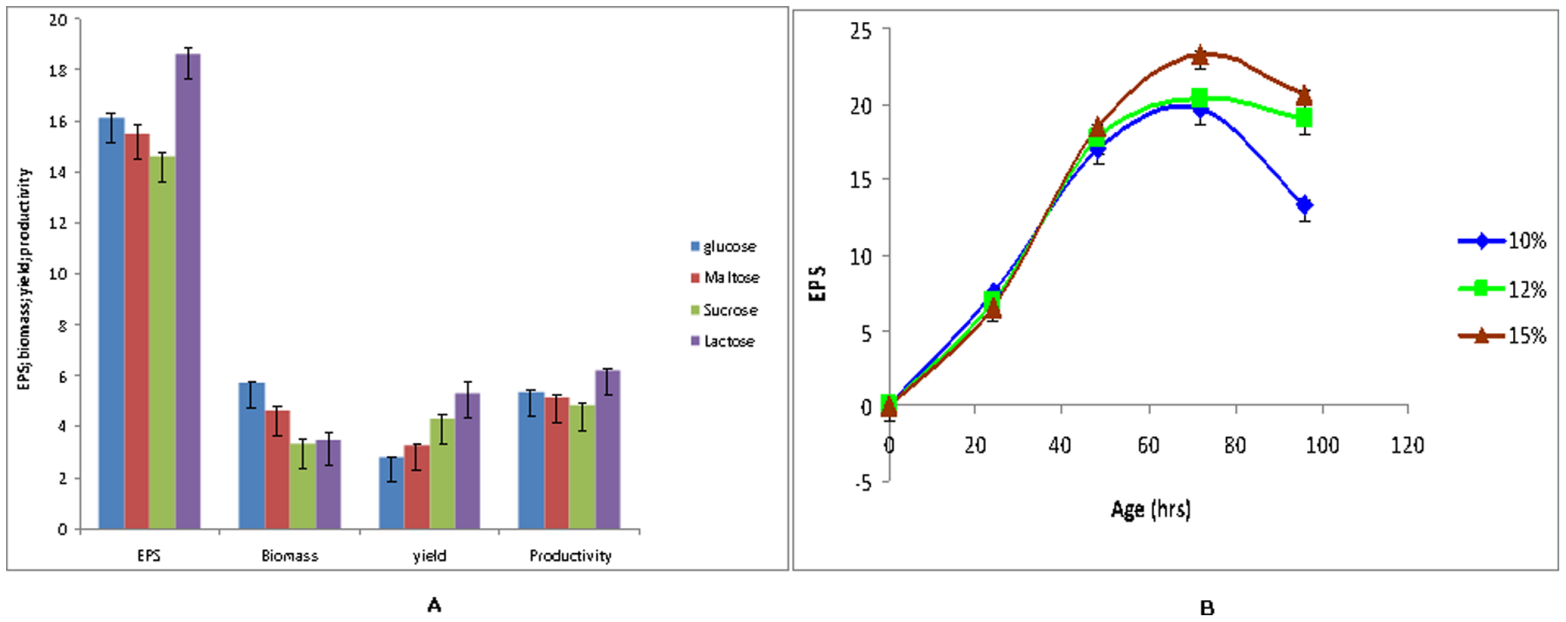
Fermentative production of exopolysaccharide. (A) Effect of different carbon sources on exopolysaccharide elaboration and biomass production by *Alteromonas macleodii* PA2. (B) Time course of exopolysaccharide production at different concentrations of lactose by *Alteromonas macleodii* PA2.

Most of the earlier reports on marine polysaccharide production indicated that glucose is a preferred carbon source for EPS production by *Alteromonas macleodii* [25]–[27]. However, substrates have significant influence on productivity in all fermentation processes and therefore, three different carbon sources (maltose, lactose, sucrose) along with glucose were selected to study the effect of carbon sources on EPS production by the selected strain. The results obtained indicated that productivity and yield of EPS was highest when lactose was used as carbon source and minimum in case of sucrose (Figure 2A). In the present study 18.7 gl^−1^ EPS was produced after 72 hours fermentation using a medium containing 50 gl^−1^ lactose with productivity of 6.23 gl^−1^day^−1^. This is significantly high as compared to earlier published report where productivity was only 2.4 gl^−1^day^−1^ [26]. Interestingly, glucose has supported maximum biomass growth where as it is least in case of lactose. This may be due to the fact that unlike lactose, glucose is readily assimilable and hence supported higher growth of the organism resulting in low production of the polysaccharide. It is also important to note *Alteromonas macleodii* PA 2 has capability of production of β-galactosidase enzyme (Table S1) and hence this strain can degrade lactose easily as compared to sucrose, the other disaccharide used as carbon source in present study. However, the expression of β-galactosidase enzyme is mostly regulated by feedback control mechanism [28] and depends on the concentration of “available” glucose present in the medium. These observations indicated that higher concentration of glucose will result in higher biomass growth and lower EPS production and on the other hand higher EPS production will be supported by a suboptimal concentration of glucose in the medium. Similar observation was also reported by Raugenes et al [26], where most of the EPS was produced during stationary phase, when the glucose concentration in the media got significantly depleted. Therefore, it may be commented that, that EPS is a product of secondary metabolism by *A.macleodii* and hence a critical concentration of glucose in the medium would promote production of EPS over biomass growth and lactose will be a better choice as carbon source than all other selected carbon sources for batch fermentation process for production of this polysaccharide.

### Effect of different concentrations of lactose

There are quite a large number of publications regarding fermentative production of marine exopolysaccharides in the literature [3], [5], [29], however, in all those cases maximally 5% sugar was used in the production media. Moreover, earlier reports on microbial polysaccharide production have shown that higher glucose concentrations have inhibitory effect on polysaccharide production by *A. macleodii* [27], [29]. On the contrary, for economically feasible production of polysaccharides, higher osmotolerance of the producing strain is very much desirable and one of the important criteria for the selection of organisms. Therefore, it was interesting to understand the effect of higher concentrations of lactose on EPS production by the selected strain. The lactose concentration was varied from 5% (w/v) to 15% (w/v) and results clearly indicated that the strain was able to grow and produce EPS at higher concentrations of lactose (Figure 2B). The growth of selected strain was found to be inhibited when 20% (w/v) lactose was used in media. EPS elaboration by the strain *Alteromonas macleodii* PA 2 has increased with increasing concentrations of lactose from 5% to 15% (w/v) and it was possible to obtain 23.4 gl^−1^ EPS after 72 hours of fermentation. This is almost 4 times higher as compared to earlier published report [26]. A comparative study of EPS elaboration, biomass production, productivity and yield after 72 hours of fermentation, showed that, higher lactose concentration has not only supported high EPS production but also it has resulted in higher productivity and yield of the polymer (Table 2). This may lead to development of economically feasible bioprocess for EPS production by *Alteromonas macleodii* PA2.

**Table 2:** EPS elaboration and biomass production at variable concentrations of lactose at 72*Alteromonas macleodii* PA2.

Lactose concentration (w/v)	EPS (gl^−1^)	O.D(600 nm)	Productivity (gl^−1^day^−1^)	EPS Yield (gl^−1^biomass^−1^)
10%	19.60±0.09	3.42±0.05	6.50±0.03	5.73±0.05
12%	20.40±0.07	3.55±0.05	6.80±0.02	5.74±0.04
15%	23.40±0.35	3.08±0.04	7.80±0.11	7.59±0.01

### Biosynthesis and characterization of silver nanoparticles using purified EPS

**Figure 3:**
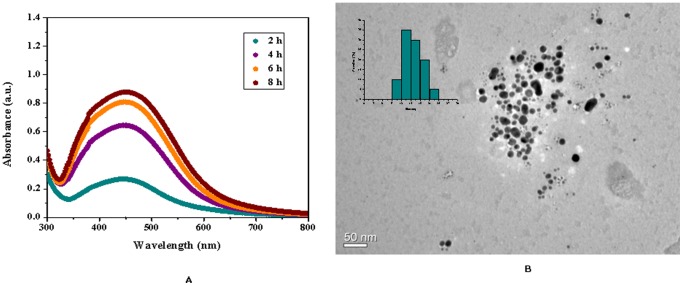
Biophysical characterization of the silver nanoparticle synthesized using the purified polysaccharide. (A) Uv-Vis spectra of sliver nanoparticles after incubation of the EPS in 9 mM solution of silver nitrate. (B) TEM image recorded from drop-coated film of AgNPs suspension after 6 hours of incubation. Scale bar corresponds to 50 nm.

**Figure 4:**
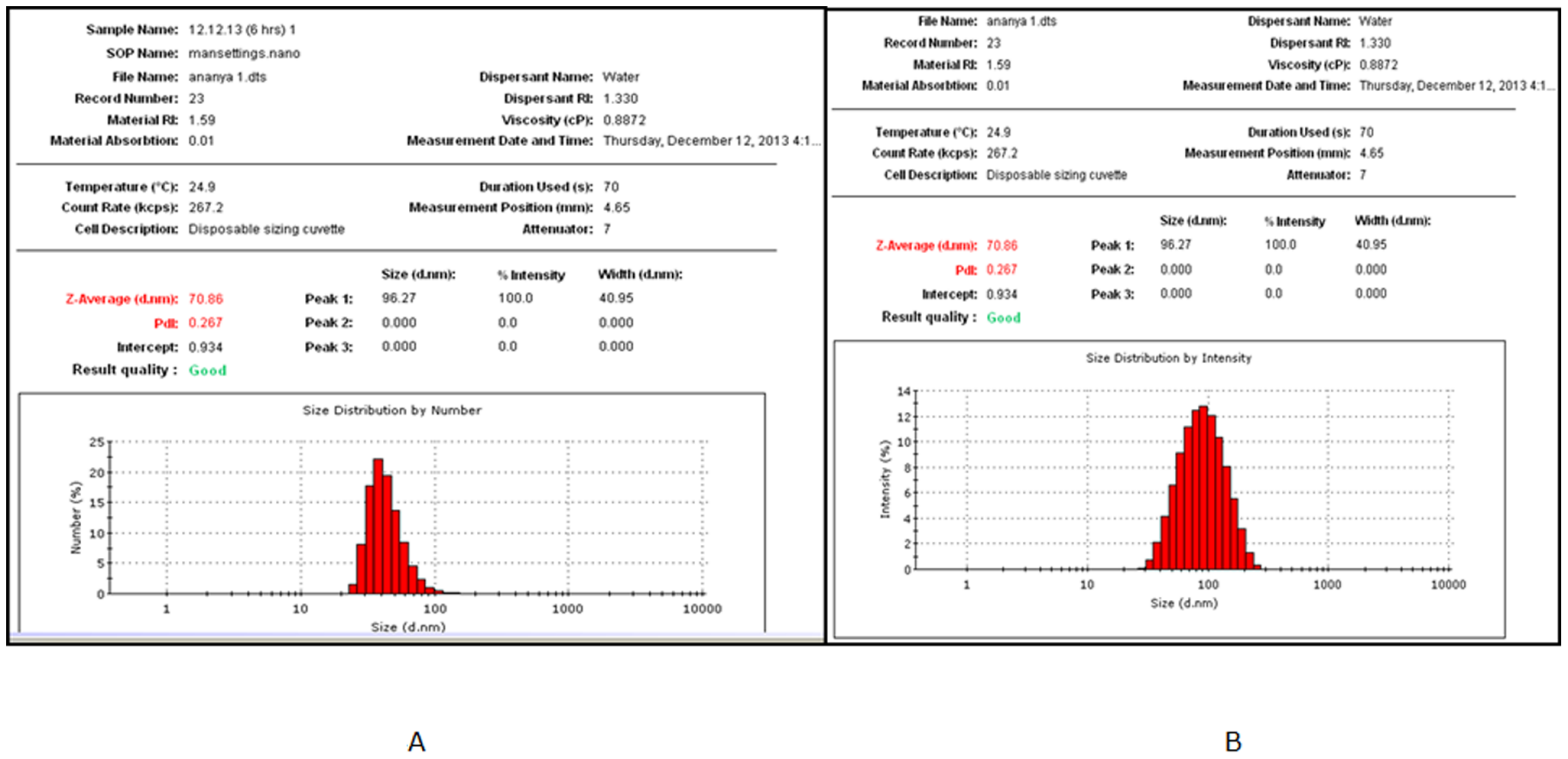
Histogram indicating size distribution by number (A) and intensity (B) of nanoparticles formed after 6 hrs of incubation.

Addition of purified polymer to silver nitrate solution in the presence of light resulted in the production of silver nanoparticles when incubated at 30°C. Formation of silver nanoparticle was initially identified by the change in colour from colourless to brick red. Further, confirmation was carried out by measuring characteristic surface plasmon resonance peak of silver nanoparticles at 430 nm by UV-visible spectrophotometer (Fig. 3A).The results were similar when compared to earlier published report of dextran stabilized silver nanoparticles [30] which suggested the formation of colloidal stable suspension of silver nanoparticles by utilizing purified polysaccharide. It is important to mention here, that the formation of silver nanoparticle initiated after 2 hours of incubation which is comparatively faster than previous reports [12], [31]. Fig. 3A illustrates that, the absorption spectra reached a plateau after 8 hours of incubation, indicating complete formation of silver nanoparticles.Morphology of the synthesized AgNPs was further studied using Transmission electron microscopy.The TEM image elucidated formation of roughly spherical shaped silver nanoparticles with less than 50 nm sizes (Fig. 3B). The histogram indicated most of the particle was in the size range of 12 to 20 nm. In contrast when silver nanoparticle was characterized by DLS the average particle size appeared to be 87.7 nm after 2 hours of incubation reduced to 70 nm after 6 hours of incubation and then further stabilizedindicating that it was possible to obtain stable colloidal suspension of the silver nanoparticles (Fig. 4).During DLS measurement the size of nanoparticles seems to be larger in comparison to TEM imaging. This may be due to the fact that during DLS measurement overlapping of nanoparticles occurred which resulted in development of electrical double layer along with interference on charged particles [32]. Further, results obtained from DLS experiments indicated that the colloidal suspension of silver nanoparticles produced were having low poly dispersity index (PDI), where, PDI values were found to remain almost unchanged during the course of incubation. The PDI value of the silver nanoparticle solution was found to be 0.265 after 2 hours of incubation and it was 0.267 after six hours of incubation, suggesting narrow particle size distribution in the colloidal suspensions. This observation was similar to the observations made by Kaleret.al., where formation of monodisperse silver nanoparticle solutionswith a PDI of 0.34 was accomplished by *Saccharomyces boulardii* [33]. Although, most of the earlier literature on synthesis of silver nanoparticles using polysaccharides indicated formation of polydisperse solution of nanoparticles [12], however, in the present study it was possible to obtain colloidal suspension of silver nanoparticles having narrow particle size distribution with reasonably low PDI values suggesting that this EPS has a better reducing and stabilizing properties and may be successfully used for green synthesis of biocompatible silver nanoparticles.

### FT-IR analysis of the exopolysaccharide and the nanoparticles formed

**Figure 5:**
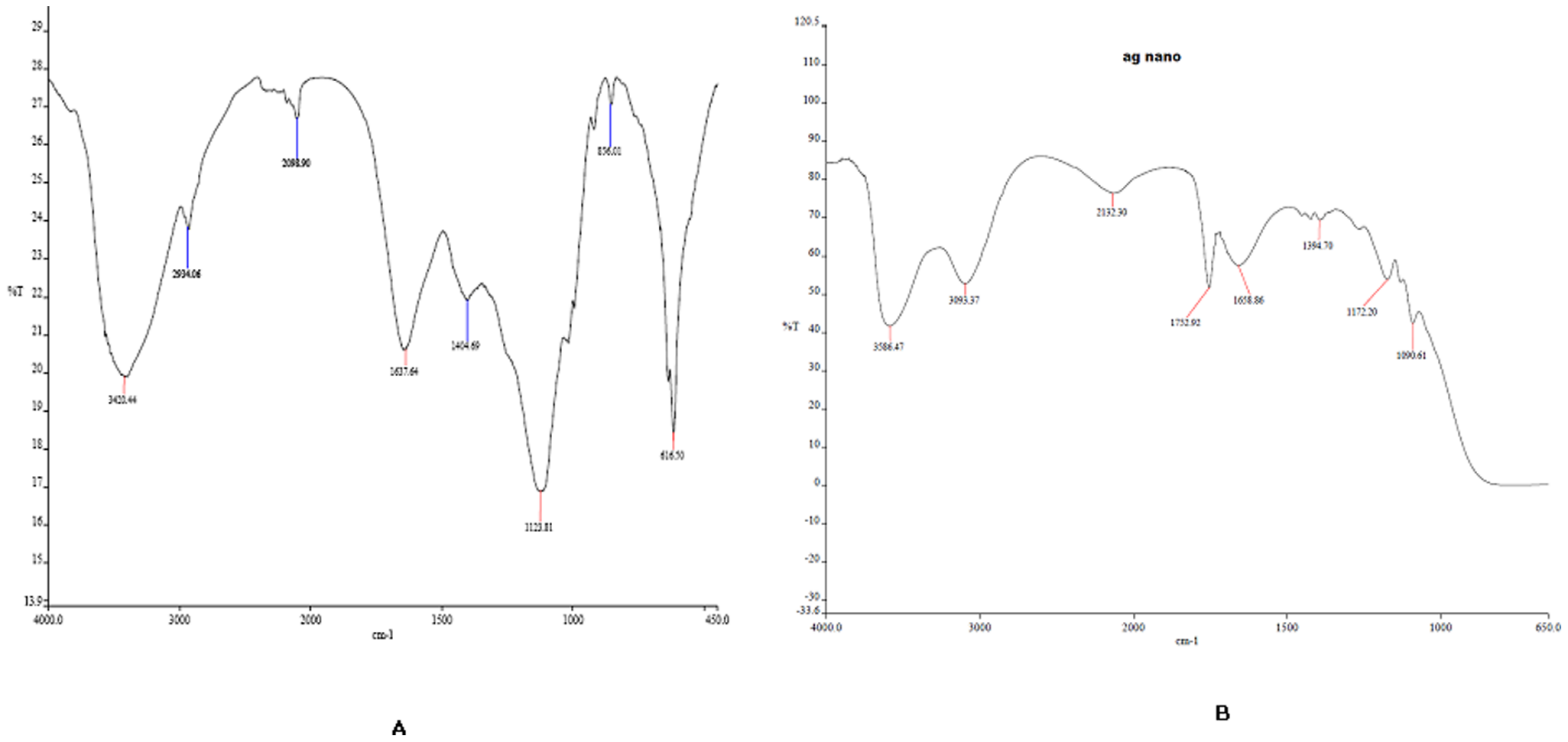
FT-IR analysis to decipher mechanism of nanoparticle biosynthesis. (A) FT-IR spectra of the polysaccharide produced by *A.macleodii* PA2 (B) FT-IR spectra of the AgNP produced and stabilized by the polysaccharide.

FT-IR spectrum of the EPS was analysed to understand the structure and presence of different functional groups in the polymer (Fig. 5A). The spectrum has revealed several characteristic peaks in the range of 3420 cm^−1^ to 800 cm^−1^. The strong absorption at 3420 cm^−1^ may be attributed to the stretching vibration of O-H group, whereas the absorption at 1673 cm^−1^ is assigned to the presence of carboxylic group. Absorption for aliphatic C-H bending was observed at 1404.69 cm^−1^. Characteristic absorption spectra in the range of 1500 cm^−1^ to 600 cm^−1^ confirmed that the molecule is a bacterial polysaccharide and is composed of sugar moieties connected with β-glycosidic linkages, as indicated by strong absorption at 856 cm^−1^ [26]. Where as in case of the FT-IR spectra of the silver nanoparticles formed (Fig. 5B), the absorption of the characteristic β-glycosidic linkages at 856 cm^−1^ was absent indicating probable involvement of this linkage towards formation of silver nanoparticle. Moreover, a new peak was observed in 1752 cm^−1^ which may be due to oxidation of the hydroxyl groups present in the EPS and simultaneous reduction of silver ions [31]. Minor shifts were observed in most of the absorption peaks, which indicate interaction between the polysaccharide and silver ions which has led to formation and stabilization of silver nanoparticles.

## Conclusions

The potential of marine microbes for commercial production of polysaccharides is still relatively less explored. The present work emphasized on the possibility of using osmotolerantmarine bacterial strain for polysaccharide production and subsequent application of that polysaccharide for the production of silver nanoparticles. Screening of several marine strains has resulted in the isolation of a novel strain of *Alteromonas macleodii*. The strain was capable of producing 23.4 gl^−1^ exopolysaccharide when 15% (w/v) lactose was used as substrate, which is considerably higher than earlier reported marine microbial polysaccharides. More importantly, the purified polymer was found to act as reducing as well as stabilizing agent for production of stable suspension of spherical silver nanoparticles with 50 nm size. Therefore, the present work highlighted to the potential of the organism for commercial exploitation towards production of this polysaccharide and further use of the same for green synthesis of biogenic silver nanoparticles.

## Supporting Information

File S1 **File includes Tables S1 and S2.** Table S1: Biochemical and enzymatic characteristics revealed from the GN cards of VITEK 2 system (bioMérieux). Table S2: Biochemical characteristics of the strain PA2.
(DOCX)
