# Supplementary material for: Extracellular Polysaccharide Production by a Novel Osmotolerant Marine Strain of Alteromonas macleodii and Its Application towards Biomineralization of Silver
Source: PLoS One. 2014 Jun 16;9(6):e98798. doi: 10.1371/journal.pone.0098798 (PMC4059621; doi:10.1371/journal.pone.0098798)
Supplement: File S1 — File includes Tables S1 and S2. Table S1: Biochemical and enzymatic characteristics revealed from the GN cards of VITEK 2 system (bioMérieux). Table S2: Biochemical characteristics of the strain PA2. (DOCX) [file pone.0098798.s001.docx]

**Supporting Information**

**Table S1: Biochemical and enzymatic characteristics revealed from the GN cards of VITEK 2 system (bioMérieux).**

| Vitek 2 GN tests | PA2 |
| --- | --- |
| Ala-Phe-Pro-arylamidase | + |
| adonitol | - |
| L-Pyrrolydonyl-arylamidase | - |
| L-Arabitol | - |
| D-Cellobiose | - |
| *β*-galactosidase | + |
| H_2_S production | - |
| *β*-*N*-acetylglucosaminidase | - |
| glutamyl arylamidase pNA | + |
| D-glucose | + |
| γ-glutamyl-transferase | - |
| Fermentation Glucose | - |
| *β*-glucosidase | + |
| D-Maltose | + |
| D-Mannitol | - |
| D-Mannose | - |
| *β*-xylosidase | - |
| *β*-alanine arylamidase pNA | - |
| L-Proline arylamidase | - |
| Lipase | + |
| Palatinose | - |
| tyrosine arylamidase | + |
| Urease | + |
| D-Sorbitol | - |
| D-Trehalose | - |
| Citrate(Sodium) | - |
| Malonate | - |
| 5-Keto-D-Gluconate | - |
| L-Lactate Alkalinisation | - |
| α-glucosidase | + |
| Succinate Alkalinisation | - |
| *β*-*N*-acetylgalactosaminidase | - |
| *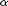*-galactosidase | + |
| Phosphatase | + |
| L-Histidine assimilation | - |
| Courmarate | - |
| *β-*glucoronidase | - |
| 0/129 Resistance | - |
| ELLMAN | + |
| L-Lactate assimilation | - |

**Table S2: Biochemical characteristics of the strain PA2**

| **Tests** | **PA2** |
| --- | --- |
| Citrate | _ |
| Gelatin hydrolysis | _ |
| Starch hydrolysis | + |
| Esculin hydrolysis | + |
| Catalase test | + |
| Oxidase test | + |
| **Acid Production from** |  |
| Fructose | + |
| Raffinose | - |
| Salicin | - |
| Xylose | - |
| Maltose | + |
| Arabinose | + |
| Mannose | - |
| Trehalose | - |
| Sucrose | + |
| Cellobiose | + |
| Glucose | + |
| Inositol | - |
| Melibiose | + |
| Dulcitol | - |
| Galactose | + |
| Sorbitol | + |

+: Positive; -: Negative
